# Supplementary figures and images for: Molecular signature to predict quality of life and survival with glioblastoma using Multiview omics model
Source: PLoS One. 2023 Nov 16;18(11):e0287448. doi: 10.1371/journal.pone.0287448 (PMC10653472; doi:10.1371/journal.pone.0287448)

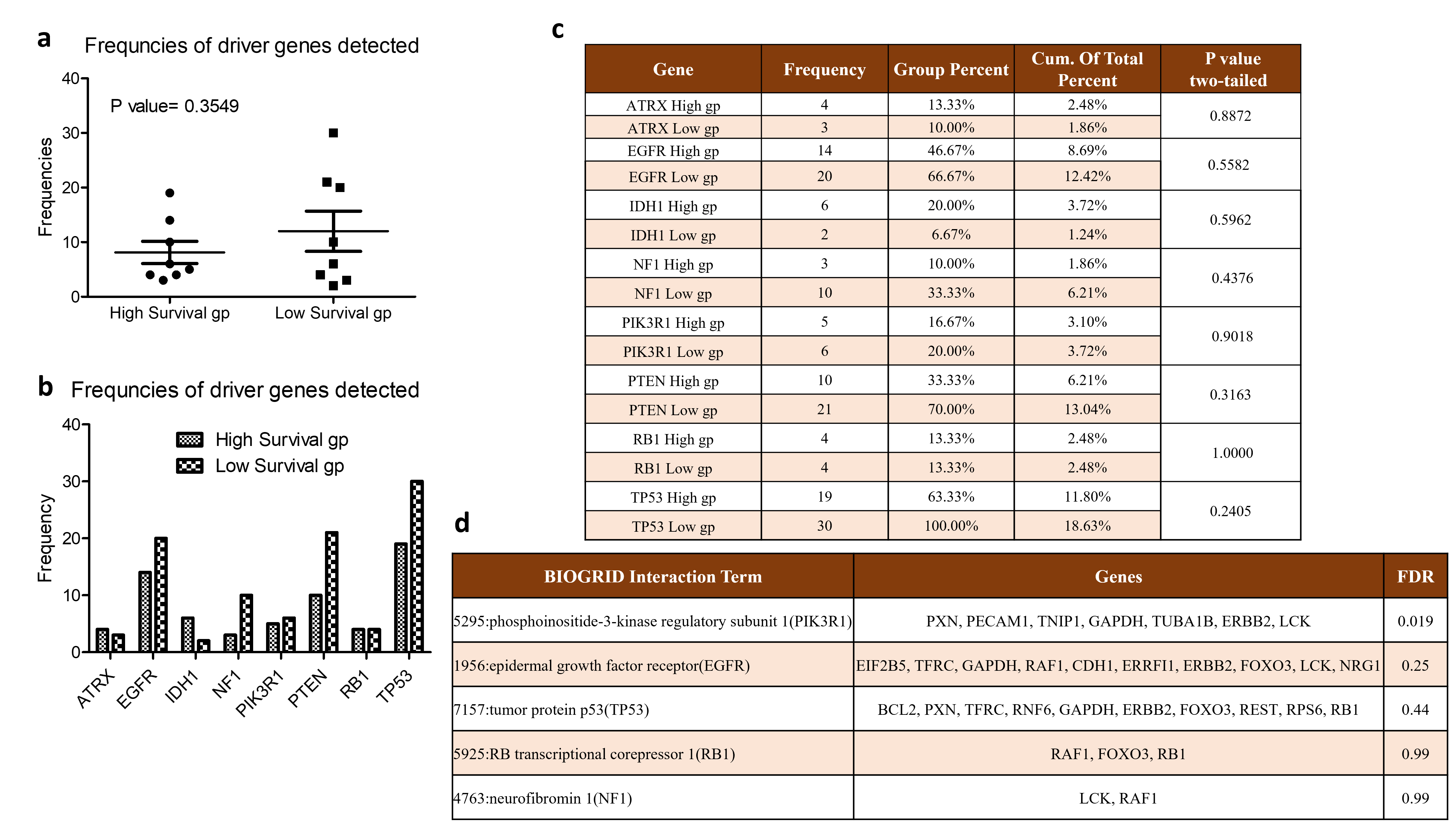

Supplement: S3 Fig — a Box plot compares frequencies of driver mutations between high and low survival groups. b bar plot compares frequencies of driver mutations in high and low survival groups. c The table shows data behind Fig a and b. d The table shows the BIOGRID interactions of MiRF predicted genes with the driver genes. A p-value of < 0.05 was considered significant. (TIF) [file pone.0287448.s003.tif]
