# Supplementary material for: Molecular signature to predict quality of life and survival with glioblastoma using Multiview omics model
Source: PLoS One. 2023 Nov 16;18(11):e0287448. doi: 10.1371/journal.pone.0287448 (PMC10653472; doi:10.1371/journal.pone.0287448)
Supplement: S1 File — (DOCX) [file pone.0287448.s004.docx]

**Supplements**

**S1a Table. Important features for overall survival (OS) ≥ 2 years.**

| SYMBOL | ENTREZ-ID | GENE NAME HOMO SAPIENS |
| --- | --- | --- |
| FAM172A | 83989 | Family with sequence similarity 172 member A(FAM172A) |
| ZKSCAN3 | 80317 | Zinc finger with KRAB and SCAN domains 3(ZKSCAN3) |
| FKBP6 | 8468 | FKBP prolyl isomerase family member 6 (inactive)(fkbp6) |
| TRIM62 | 55223 | Tripartite motif containing 62(TRIM62) |
| REST | 5978 | RE1 silencing transcription factor(rest) |
| NOL3 | 8996 | Nucleolar protein 3(NOL3) |
| CRELD1 | 78987 | Cysteine rich with EGF like domains 1(CRELD1) |
| DRG2 | 1819 | Developmentally regulated GTP binding protein 2(DRG2) |
| TNIP1 | 10318 | TNFAIP3 interacting protein 1(TNIP1) |
| B3GAT3 | 26229 | Beta-1,3-glucuronyltransferase 3(B3GAT3) |
| AGFG2 | 3268 | Arfgap with FG repeats 2(AGFG2) |
| NCKIPSD | 51517 | NCK interacting protein with SH3 domain(nckipsd) |
| GAPDH | 2597 | Glyceraldehyde-3-phosphate dehydrogenase(gapdh) |
| TFRC | 7037 | Transferrin receptor(tfrc) |
| NRG1/Heregulin | 3084 | Neuregulin 1(NRG1) |
| PXN/Paxillin | 5829 | Paxillin(pxn) |
| G6PD | 2539 | Glucose-6-phosphate dehydrogenase(g6pd) |
| BCL2 | 596 | BCL2 apoptosis regulator(bcl2) |
| ERRFI1/MIG.6 | 54206 | ERBB receptor feedback inhibitor 1(ERRFI1) |
| RAF1/CRaf | 5894 | Raf-1 proto-oncogene, serine/threonine kinase(raf1) |
| FOXO3/FOXO3a | 2309 | Forkhead box O3(FOXO3) |
| EEF2K | 29904 | Eukaryotic elongation factor 2 kinase(eef2k) |

| SYMBOL | ENTREZ-ID | GENE NAME HOMO SAPIENS |
| --- | --- | --- |
| SERPINB10 | 5273 | Serpin family B member 10(SERPINB10) |
| TM4SF20 | 79853 | Transmembrane 4 L six family member 20(TM4SF20) |
| POU2F3 | 25833 | POU class 2 homeobox 3(POU2F3) |
| EIF2B5 | 8893 | Eukaryotic translation initiation factor 2B subunit epsilon(eif2b5) |
| MLN | 4295 | Motilin(mln) |
| WRNIP1 | 56897 | WRN helicase interacting protein 1(WRNIP1) |
| GALK1 | 2584 | Galactokinase 1(GALK1) |
| RNF121 | 55298 | Ring finger protein 121(RNF121) |
| RNF6 | 6049 | Ring finger protein 6(RNF6) |
| TFRC.TFRC | 7037 | Transferrin receptor(tfrc) |
| TUBA1B/Acetyl.a.Tubulin.Lys40 | 10376 | Tubulin alpha 1b(tuba1b) |
| ERBB2/HER2 | 2064 | Erb-b2 receptor tyrosine kinase 2(ERBB2) |
| RPS6/S6_pS240_S244 | 6194 | Ribosomal protein S6(RPS6) |
| PECAM1/CD31 | 5175 | Platelet and endothelial cell adhesion molecule 1(PECAM1) |
| LCK | 3932 | LCK proto-oncogene, src family tyrosine kinase(lck) |
| NDRG1/NDRG1_pT346 | 10397 | N-myc downstream regulated 1(NDRG1) |
| CDH1/E-Cadherin | 999 | Cadherin 1(CDH1) |
| FOXO3/FOXO3a_pS318_S321 | 2309 | Forkhead box O3(FOXO3) |
| RB1/Rb_pS807_S811 | 5925 | RB transcriptional corepressor 1(RB1) |

**S1b Table: Important features for quality of life (QOL) ≥ 80 KPS.**

| GO-BP Term  **S2a Table: Gene Ontology-Biological processes for MiRF predicted features.** | Genes | FDR |
| --- | --- | --- |
| GO:0002931~response to ischemia | BCL2, NOL3, EEF2K, REST | 0.111627 |
| GO:0043066~negative regulation of apoptotic process | BCL2, NOL3, TFRC, RAF1, ERBB2, EEF2K, RPS6 | 0.126466 |
| GO:0043065~positive regulation of apoptotic process | EIF2B5, BCL2, FOXO3, REST, RPS6 | 0.549955 |
| GO:0071466~cellular response to xenobiotic stimulus | TFRC, REST, RB1 | 0.549955 |
| GO:0038133~ERBB2-ERBB3 signaling pathway | ERBB2, NRG1 | 0.549955 |
| GO:0001934~positive regulation of protein phosphorylation | PECAM1, TFRC, RAF1, ERBB2 | 0.549955 |
| GO:0051402~neuron apoptotic process | BCL2, GAPDH, RB1 | 0.549955 |
| GO:0038135~ERBB2-ERBB4 signaling pathway | ERBB2, NRG1 | 0.549955 |
| GO:0035556~intracellular signal transduction | TFRC, RAF1, ERBB2, LCK, NRG1 | 0.549955 |
| GO:0045893~positive regulation of transcription, DNA-templated | TRIM62, RNF6, CDH1, ZKSCAN3, FOXO3, REST | 0.549955 |
| GO:0009410~response to xenobiotic stimulus | BCL2, CDH1, FOXO3, LCK | 0.549955 |
| GO:0033138~positive regulation of peptidyl-serine phosphorylation | BCL2, TFRC, RAF1 | 0.549955 |
| GO:0030307~positive regulation of cell growth | BCL2, ERBB2, NRG1 | 0.549955 |
| GO:0010468~regulation of gene expression | BCL2, NOL3, CDH1, REST | 0.549955 |
| GO:0042060~wound healing | RAF1, ERBB2, NRG1 | 0.549955 |
| GO:0044314~protein K27-linked ubiquitination | TRIM62, RNF6 | 0.567381 |
| GO:0032869~cellular response to insulin stimulus | ERRFI1, EEF2K, RB1 | 0.567381 |
| GO:0043922~negative regulation by host of viral transcription | POU2F3, REST | 0.76854 |
| GO:0071456~cellular response to hypoxia | BCL2, NDRG1, FOXO3 | 0.76854 |
| GO:0007169~transmembrane receptor protein tyrosine kinase signaling pathway | ERBB2, LCK, NRG1 | 0.76854 |
| GO:0042551~neuron maturation | BCL2, RB1 | 0.774626 |
| GO:0010039~response to iron ion | BCL2, TFRC | 0.774626 |
| GO:0007165~signal transduction | PXN, MLN, PECAM1, RAF1, DRG2, ERBB2, NDRG1 | 0.774626 |
| GO:0030330~DNA damage response, signal transduction by p53 class mediator | NDRG1, FOXO3 | 0.774776 |
| GO:0007568~aging | EIF2B5, TFRC, FOXO3 | 0.793444 |
| GO:0001666~response to hypoxia | NOL3, TFRC, REST | 0.811583 |
| GO:0014032~neural crest cell development | FAM172A, NRG1 | 0.811583 |
| GO:0030154~cell differentiation | FKBP6, RAF1, LCK, NRG1, RB1 | 0.831685 |
| GO:0030517~negative regulation of axon extension | RNF6, CDH1 | 0.831685 |
| GO:2000378~negative regulation of reactive oxygen species metabolic process | BCL2, G6PD | 0.840663 |
| GO:0097150~neuronal stem cell population maintenance | FOXO3, REST | 0.849015 |
| GO:0030336~negative regulation of cell migration | BCL2, CDH1, FOXO3 | 0.891647 |
| GO:2001243~negative regulation of intrinsic apoptotic signaling pathway | BCL2, NOL3 | 0.906586 |
| GO:0098609~cell-cell adhesion | BCL2, PECAM1, CDH1 | 0.906586 |
| GO:0043123~positive regulation of I-kappaB kinase/NF-kappaB signaling | TRIM62, TFRC, GAPDH | 0.91278 |
| GO:0043085~positive regulation of catalytic activity | BCL2, B3GAT3 | 0.91278 |
| GO:0007159~leukocyte cell-cell adhesion | PECAM1, TNIP1 | 0.926187 |
| GO:2001234~negative regulation of apoptotic signaling pathway | BCL2, RB1 | 0.926187 |
| GO:0097192~extrinsic apoptotic signaling pathway in absence of ligand | BCL2, FOXO3 | 0.933819 |
| GO:0048709~oligodendrocyte differentiation | ERBB2, NRG1 | 0.933819 |
| GO:2001240~negative regulation of extrinsic apoptotic signaling pathway in absence of ligand | BCL2, NRG1 | 0.951889 |
| GO:0098742~cell-cell adhesion via plasma-membrane adhesion molecules | PECAM1, CDH1 | 0.951889 |
| GO:0033077~T cell differentiation in thymus | BCL2, RPS6 | 0.951889 |
| GO:0008625~extrinsic apoptotic signaling pathway via death domain receptors | BCL2, RAF1 | 0.951889 |
| GO:0010628~positive regulation of gene expression | TFRC, ERBB2, REST, NRG1 | 0.951889 |
| GO:0045944~positive regulation of transcription from RNA polymerase II promoter | TNIP1, RAF1, POU2F3, FOXO3, REST, RB1 | 0.954111 |
| GO:0071364~cellular response to epidermal growth factor stimulus | ERRFI1, ERBB2 | 0.977976 |
| GO:0008284~positive regulation of cell proliferation | BCL2, ERBB2, NRG1, RPS6 | 0.9813 |
| GO:0030890~positive regulation of B cell proliferation | BCL2, TFRC | 0.9813 |
| GO:0048538~thymus development | BCL2, RAF1 | 0.982811 |
| GO:0042552~myelination | EIF2B5, ERBB2 | 1 |
| GO:0010507~negative regulation of autophagy | BCL2, ZKSCAN3 | 1 |
| GO:0031334~positive regulation of protein complex assembly | TFRC, NRG1 | 1 |
| GO:0035019~somatic stem cell population maintenance | RAF1, REST | 1 |
| GO:0045892~negative regulation of transcription, DNA-templated | ZKSCAN3, REST, NRG1, RB1 | 1 |
| GO:0010951~negative regulation of endopeptidase activity | SERPINB10, GAPDH | 1 |
| GO:2000134~negative regulation of G1/S transition of mitotic cell cycle | BCL2, RB1 | 1 |
| GO:0042149~cellular response to glucose starvation | BCL2, FOXO3 | 1 |
| GO:0045785~positive regulation of cell adhesion | ERBB2, NRG1 | 1 |
| GO:0006915~apoptotic process | BCL2, NOL3, RAF1, FOXO3 | 1 |
| GO:0006006~glucose metabolic process | GAPDH, G6PD | 1 |
| GO:0045665~negative regulation of neuron differentiation | FOXO3, REST | 1 |
| GO:0000082~G1/S transition of mitotic cell cycle | BCL2, RPS6 | 1 |
| GO:0000381~regulation of alternative mRNA splicing, via spliceosome | FAM172A, REST | 1 |
| GO:0007416~synapse assembly | CDH1, NRG1 | 1 |
| GO:0009636~response to toxic substance | BCL2, CDH1 | 1 |
| GO:0070373~negative regulation of ERK1 and ERK2 cascade | TNIP1, ERRFI1 | 1 |
| GO:0034599~cellular response to oxidative stress | FOXO3, G6PD | 1 |
| GO:0002181~cytoplasmic translation | DRG2, RPS6 | 1 |
| GO:0000902~cell morphogenesis | CDH1, NRG1 | 1 |
| GO:0031175~neuron projection development | CDH1, RB1 | 1 |
| GO:0030308~negative regulation of cell growth | BCL2, RB1 | 1 |
| GO:0008285~negative regulation of cell proliferation | RAF1, NDRG1, REST | 1 |
| GO:0050853~B cell receptor signaling pathway | BCL2, LCK | 1 |
| GO:0000226~microtubule cytoskeleton organization | GAPDH, TUBA1B | 1 |
| GO:0050852~T cell receptor signaling pathway | EIF2B5, LCK | 1 |
| GO:0018108~peptidyl-tyrosine phosphorylation | ERBB2, LCK | 1 |
| GO:0006468~protein phosphorylation | RAF1, ERBB2, LCK | 1 |
| GO:0007275~multicellular organism development | CDH1, ERBB2 | 1 |
| GO:0016567~protein ubiquitination | TRIM62, RNF121, RNF6 | 1 |
| GO:0000165~MAPK cascade | RAF1, NRG1 | 1 |
| GO:0043410~positive regulation of MAPK cascade | RAF1, ERBB2 | 1 |
| GO:0051092~positive regulation of NF-kappaB transcription factor activity | TRIM62, TFRC | 1 |
| GO:0042127~regulation of cell proliferation | ERBB2, NDRG1 | 1 |
| GO:0007156~homophilic cell adhesion via plasma membrane adhesion molecules | PECAM1, CDH1 | 1 |
| GO:0000122~negative regulation of transcription from RNA polymerase II promoter | ZKSCAN3, FOXO3, REST, RB1 | 1 |
| GO:0046777~protein autophosphorylation | ERBB2, EEF2K | 1 |
| GO:0008283~cell proliferation | BCL2, NRG1 | 1 |
| GO:0008380~RNA splicing | FAM172A, NOL3 | 1 |
| GO:0045087~innate immune response | TRIM62, WRNIP1, LCK | 1 |
| GO:0006351~transcription, DNA-templated | PXN, NRG1 | 1 |
| GO:0006412~translation | TNIP1, RPS6 | 1 |
| GO:0042981~regulation of apoptotic process | NOL3, RAF1 | 1 |
| GO:0006357~regulation of transcription from RNA polymerase II promoter | TNIP1, ZKSCAN3, POU2F3, FOXO3, RB1 | 1 |
| GO:0010629~negative regulation of gene expression | REST, RB1 | 1 |
| GO:0007166~cell surface receptor signaling pathway | PECAM1, ERBB2 | 1 |
| GO:0051301~cell division | TUBA1B, RB1 | 1 |
| GO:0006355~regulation of transcription, DNA-templated | RNF6, REST, RB1 | 1 |

| **KEGG Term**  **S2b Table: KEGG Pathways for MiRF predicted features.** | **Genes** | **FDR** | **References** |
| --- | --- | --- | --- |
| hsa01521: EGFR tyrosine kinase inhibitor resistance | BCL2, RAF1, ERBB2, FOXO3, NRG1, RPS6 | 0.000314 | [1, 2], [3], [4], [5], [6], [7, 8] |
| hsa05219: Bladder cancer | RAF1, CDH1, ERBB2, RB1 | 0.010525 |  |
| hsa04066: HIF-1 signaling pathway | BCL2, TFRC, GAPDH, ERBB2, RPS6 | 0.010525 | [9],[10], [11], [12], [13] |
| hsa05213: Endometrial cancer | RAF1, CDH1, ERBB2, FOXO3 | 0.019733 |  |
| hsa05226: Gastric cancer | BCL2, RAF1, CDH1, ERBB2, RB1 | 0.02078 |  |
| hsa05223: Non-small cell lung cancer | RAF1, ERBB2, FOXO3, RB1 | 0.024785 |  |
| hsa05215: Prostate cancer | BCL2, RAF1, ERBB2, RB1 | 0.045208 |  |
| hsa01522: Endocrine resistance | BCL2, RAF1, ERBB2, RB1 | 0.045208 | [14, 15] |
| hsa04151: PI3K-Akt signaling pathway | BCL2, RAF1, ERBB2, FOXO3, RPS6 | 0.198352 | [7, 8] |
| hsa05230: Central carbon metabolism in cancer | RAF1, ERBB2, G6PD | 0.198352 | [16], [17] |
| hsa05218: Melanoma | RAF1, CDH1, RB1 | 0.198352 |  |
| hsa04510: Focal adhesion | BCL2, PXN, RAF1, ERBB2 | 0.198352 | [18-20] |
| hsa05205: Proteoglycans in cancer | PXN, RAF1, ERBB2, RPS6 | 0.198352 | [21-23] |
| hsa05212: Pancreatic cancer | RAF1, ERBB2, RB1 | 0.198352 |  |
| hsa04012: ErbB signaling pathway | RAF1, ERBB2, NRG1 | 0.228395 | [24-26], [27] |
| hsa05131: Shigellosis | BCL2, PXN, TNIP1, FOXO3 | 0.270127 |  |
| hsa05132: Salmonella infection | BCL2, GAPDH, RAF1, TUBA1B | 0.270127 |  |
| hsa04722: Neurotrophin signaling pathway | BCL2, RAF1, FOXO3 | 0.35346 | [28] |
| hsa04210: Apoptosis | BCL2, RAF1, TUBA1B | 0.403238 | [1, 2],[4],[29-31] |
| hsa04371: Apelin signaling pathway | RAF1, CDH1, RPS6 | 0.403238 | [32-34] |
| hsa05200: Pathways in cancer | BCL2, RAF1, CDH1, ERBB2, RB1 | 0.403238 | [7, 8] |
| hsa05224: Breast cancer | RAF1, ERBB2, RB1 | 0.421714 |  |
| hsa04218: Cellular senescence | RAF1, FOXO3, RB1 | 0.447667 | [31, 35] |
| hsa05161: Hepatitis B | BCL2, RAF1, RB1 | 0.458141 |  |
| hsa04062: Chemokine signaling pathway | PXN, RAF1, FOXO3 | 0.588213 | [36, 37] |
| hsa05170: Human immunodeficiency virus 1 infection | BCL2, PXN, RAF1 | 0.642635 |  |
| hsa05207: Chemical carcinogenesis - receptor activation | BCL2, RAF1, RB1 | 0.642635 | [38] |
| hsa05163: Human cytomegalovirus infection | PXN, RAF1, RB1 | 0.683325 | [39] |
| hsa04370: VEGF signaling pathway | PXN, RAF1 | 0.776211 | [7, 8] |
| hsa04917: Prolactin signaling pathway | RAF1, FOXO3 | 0.819821 | [40, 41] |
| hsa04520: Adherens junction | CDH1, ERBB2 | 0.819821 | [42, 43] |
| hsa01524: Platinum drug resistance | BCL2, ERBB2 | 0.819821 | [44] |
| hsa05214: Glioma | RAF1, RB1 | 0.819821 | [7, 8] |
| hsa05220: Chronic myeloid leukemia | RAF1, RB1 | 0.819821 |  |
| hsa05100: Bacterial invasion of epithelial cells | PXN, CDH1 | 0.819821 |  |
| hsa05206: MicroRNAs in cancer | BCL2, RAF1, ERBB2 | 0.837224 | [45] |
| hsa05210: Colorectal cancer | BCL2, RAF1 | 0.837224 |  |
| hsa04540: Gap junction | RAF1, TUBA1B | 0.837224 | [42] |
| hsa05235: PD-L1 expression and PD-1 checkpoint pathway in cancer | RAF1, LCK | 0.837224 | [46-49] |
| hsa05222: Small cell lung cancer | BCL2, RB1 | 0.838025 |  |
| hsa05165: Human papillomavirus infection | PXN, RAF1, RB1 | 0.838025 |  |
| hsa04660: T cell receptor signaling pathway | RAF1, LCK | 0.838025 | [50-52] |
| hsa04064: NF-kappa B signaling pathway | BCL2, LCK | 0.838025 | [7, 53] |
| hsa04928: Parathyroid hormone synthesis, secretion and action | BCL2, RAF1 | 0.838025 | [54, 55] |
| hsa05014: Amyotrophic lateral sclerosis | BCL2, TUBA1B, NRG1 | 0.838025 | [56, 57] |
| hsa04670: Leukocyte transendothelial migration | PXN, PECAM1 | 0.838025 | [43, 58, 59] |
| hsa01200: Carbon metabolism | GAPDH, G6PD | 0.838025 | [16], [17], [11, 60] |
| hsa04071: Sphingolipid signaling pathway | BCL2, RAF1 | 0.838025 | [61, 62] |
| hsa04152: AMPK signaling pathway | EEF2K, FOXO3 | 0.838025 | [63-65] |
| hsa05010: Alzheimer disease | GAPDH, RAF1, TUBA1B | 0.838025 | [66-69] |
| hsa04650: Natural killer cell mediated cytotoxicity | RAF1, LCK | 0.838025 | [70, 71] |
| hsa04068: FoxO signaling pathway | RAF1, FOXO3 | 0.838025 | [72-75] |
| hsa05135: Yersinia infection | PXN, LCK | 0.838025 |  |
| hsa04910: Insulin signaling pathway | RAF1, RPS6 | 0.838025 | [76-78] |
| hsa04915: Estrogen signaling pathway | BCL2, RAF1 | 0.838025 | [79, 80] |
| hsa05418: Fluid shear stress and atherosclerosis | BCL2, PECAM1 | 0.838025 | [81-83] |
| hsa04140: Autophagy - animal | BCL2, RAF1 | 0.838025 | [29, 31, 65, 84, 85] |
| hsa04550: Signaling pathways regulating pluripotency of stem cells | RAF1, REST | 0.838025 | [7, 8, 75, 86] |
| hsa04145: Phagosome | TFRC, TUBA1B | 0.838025 | [65, 87] |
| hsa04921: Oxytocin signaling pathway | RAF1, EEF2K | 0.838025 | [88, 89] |
| hsa04150: mTOR signaling pathway | RAF1, RPS6 | 0.838025 | [7, 8, 63, 64] |
| hsa04514: Cell adhesion molecules | PECAM1, CDH1 | 0.838025 | [42] |
| hsa05160: Hepatitis C | RAF1, RB1 | 0.838025 |  |
| hsa04630: JAK-STAT signaling pathway | BCL2, RAF1 | 0.848152 | [90, 91] |
| hsa05225: Hepatocellular carcinoma | RAF1, RB1 | 0.848152 |  |
| hsa04530: Tight junction | TUBA1B, ERBB2 | 0.848152 | [42, 43] |
| hsa05022: Pathways of neurodegeneration - multiple diseases | BCL2, RAF1, TUBA1B | 0.853258 | [66, 76, 92] |
| hsa05152: Tuberculosis | BCL2, RAF1 | 0.864777 |  |
| hsa05167: Kaposi sarcoma-associated herpesvirus infection | RAF1, RB1 | 0.874104 |  |
| hsa05168: Herpes simplex virus 1 infection | EIF2B5, BCL2, POU2F3 | 0.874104 |  |
| hsa05130: Pathogenic Escherichia coli infection | GAPDH, TUBA1B | 0.874104 |  |
| hsa05169: Epstein-Barr virus infection | BCL2, RB1 | 0.874104 |  |
| hsa05415: Diabetic cardiomyopathy | GAPDH, G6PD | 0.874104 | [93-96] |
| hsa05203: Viral carcinogenesis | PXN, RB1 | 0.874104 | [39] |
| hsa04015: Rap1 signaling pathway | RAF1, CDH1 | 0.88125 | [43, 97] |
| hsa05417: Lipid and atherosclerosis | BCL2, POU2F3 | 0.884883 | [98, 99] |
| hsa05166: Human T-cell leukemia virus 1 infection | LCK, RB1 | 0.885513 |  |
| hsa05208: Chemical carcinogenesis - reactive oxygen species | RAF1, FOXO3 | 0.885513 | [4, 100-102] |
| hsa04810: Regulation of actin cytoskeleton | PXN, RAF1 | 0.891241 | [103, 104] |
| hsa04010: MAPK signaling pathway | RAF1, ERBB2 | 0.97351 | [7, 8] |
| hsa05016: Huntington disease | TUBA1B, REST | 0.97351 | [66, 105, 106] |
| hsa01100: Metabolic pathways | GAPDH, GALK1, B3GAT3, G6PD | 0.97351 | [16], [17], [11, 60], [107], [108] |

**Figures legends**

**Fig 1. Multiview iterative random forests (MiRF) model.** The iRF algorithm has 3 main components: first, iterative reweighted RF with K times of iterations; second, generalized RIT that takes projected binary features from the last feature-weighted RF as input; and third, bagged stability scores that aggregate interactions that are prevalent in B bootstrapped samples. The 3 steps of bootstrapping indicated in blue. The model had 2 main outputs: important features with the highest Gini importance returned in at least 50% of bootstrapped replicates, and interactions between important features with stability scores > 0.5 were returned from bootstrapped replicates. The Multiview IRF model is composed of six models: Three models for OS include gene expression, proteomics, and integrated omics; and three for KPS include gene expression, proteomics, and integrated omics.

**Fig 2. MiRF model performance in feature prediction and interaction recovery. a** 95% CIs of mean AUPR curves for gene expression, proteomics, and integrated omics data used to predict important features in patients who lived for more than 2 years. **b** 95% CIs of mean AUROC curves for gene expression, proteomics, and integrated omics data used to recover interactions in patients who lived for more than 2 years. **c** 95% CIs of mean AUPR curves for gene expression, proteomics, and integrated omics data used to predict important features in patients who had KPS ≥ 80. **d** 95% CIs of mean AUROC curves for gene expression, proteomics, and integrated omics data used to recover interactions in patients who had KPS ≥ 80.

**Fig 3. Stability scores of recovered interactions from the MiRF model. a** Stability scores of recovered interactions for OS ≥ 2 years. **b** Stability scores of recovered interactions for QOL ≥ 80 KPS. Colors indicate interactions from different datasets. Blue indicates interactions between proteomics data, orange indicates interactions between gene expression data, and red indicates interactions between integrated omics.

**Fig 4. Functional annotation and interaction analysis. a** Clustered heatmaps illustrate the functional annotation obtained from the DAVID database grouped by the list molecular features obtained from MiRF for OS ≥ 2 years and QOL ≥ 80 KPS. Each cell reports the fold enrichment of each gene toward a specific annotation. Darker coloring is associated with larger values and vice versa. The coloring of clusters corresponds to the following annotations: red represents KEGG pathways and blue represents GO-BP annotations. **b** Pie chart represent

**c** Protein–protein interactions from the STRING database for both OS ≥ 2 years and QOL ≥ 80 KPS, respectively. Colored nodes indicate query proteins and the first shell of interactors. Interactions include known interactions from curated databases and those that were experimentally determined; predicted interactions by gene neighborhood, gene fusion, and gene co-occurrence; and others by text mining, co-expression, and protein homology. All the different interactions can be recognized by the different colors. Colored nodes represent query proteins and the first shell of interactors; filled nodes are the 3D structure of known or predicted proteins.

**Fig 5. Regularized CPH regression model. a1** and **b1** Plots of the 10-fold cross-validated error rates show the optimal lambda value with the higher Harrell C-index and minimum error for both OS and overall signatures models, respectively. **a2** and **b2** Dot charts show β values of each feature. Values that are closer to zero are the least important features, and vice versa.

**Fig 6. MiRF signatures validation. a1** and **a2** 3D PCA scatter plots with their explained variance plots for OS and QOL signatures show two clusters for each model: blue for OS ≥ 2 years, green for OS ≤ 6 months and blue for KPS ≥ 80, green for KPS ≤ 60, respectively. **b1** and **b2** Heatmaps represent internal validation on the list of molecular features obtained from MiRF for OS ≥ 2 years and QOL ≥ 80 KPS. Each cell reports the average expression of all samples for each signature. Blue shades indicate high expression and red shades indicate low expression. **c1** and **c2** t-SNE plots show the effect of OS and overall signatures, respectively, in distinguishing OS ≥ 2 years and OS ≤ 6 months groups. Each plot has two clusters have distinct expression profile of OS and overall signatures. Red for OS ≥ 2 years, green for OS ≤ 6 months. **d1** and **d2** Boxplots represent the expression mean cutoffs for OS ≥ 2 years and OS ≤ 6 months of both OS and overall signatures, respectively. Red for OS ≥ 2 years, green for OS ≤ 6 months. A p-value of < 0.05 was considered significant.

**S1 Fig. AUROC and AUPR curves.** The PR and ROC curves represent the performance of a binary classification model on bootstrapped test datasets with class imbalance (median curves with their AUCs are shown). **a1 & a2** AUPR and AUROC curves of the gene expression MiRF model for OS ≥ 2 years and KPS ≥ 80 models, respectively. **b1 & b2** AUPR and AUROC curves of the proteomics MiRF model for OS ≥ 2 years and KPS ≥ 80 models, respectively. **c1 & c2** AUPR and AUROC curves of the integrated omics MiRF model for OS ≥ 2 years and KPS ≥ 80 models, respectively. An AUC value closer to 1 indicates better performance. In AUPR curves the color scale on the right side of the plot represents the value of the threshold. This threshold is used to calculate the precision and recall values for each point on the curve. Each shade represents a different threshold value. The fact that we have small data points in the test dataset is reflected in the PR curves which display only two colors (thresholds). This means precision and recall values at different classification thresholds may not be well represented. However, we coped with this problem by bootstrapping and taking the median of model performance, and through using a second parameter AUROC. This provides a comprehensive understanding of the model's statistical performance on binary classification data with class imbalance.

**S2 Fig. Kaplan–Meier analysis. a** KM plots and log-rank test p-values of important genes obtained from the MiRF model for OS ≥ 2 years. **b** KM plots and log-rank test p-values of important proteins obtained from the MiRF model for OS ≥ 2 years. **c** KM plots and log-rank test p-values of important genes obtained from the MiRF model for KPS ≥ 80. **d** KM plots and log-rank test p-values of important proteins obtained from the MiRF model for KPS ≥ 80. **e** KM plot and log-rank test p-value of KPS. A p-value of < 0.05 was considered significant.

**S3 Fig. Mutations in the driver genes in our specified TCGA dataset. a** Box plot compares frequencies of driver mutations between high and low survival groups. **b** bar plot compares frequencies of driver mutations in high and low survival groups. **c** The table shows data behind Fig a and b. **d** The table shows the BIOGRID interactions of MiRF predicted genes with the driver genes. A p-value of < 0.05 was considered significant.

References:

1. Fels C, Schäfer C, Hüppe B, Bahn H, Heidecke V, Kramm CM, et al. Bcl-2 expression in higher-grade human glioma: a clinical and experimental study. Journal of neuro-oncology. 2000;48(3):207-16.

2. Ghobrial IM, Witzig TE, Adjei AA. Targeting apoptosis pathways in cancer therapy. CA: a cancer journal for clinicians. 2005;55(3):178-94.

3. Mineo J-F, Bordron A, Isabelle Q-R, Maurage C-A, Virginie B, Séverine L, et al. Increasing of HER2 membranar density in human glioblastoma U251MG cell line established in a new nude mice model. Journal of neuro-oncology. 2006;76(3):249-55.

4. Yao S, Fan LY-N, Lam EW-F, editors. The FOXO3-FOXM1 axis: A key cancer drug target and a modulator of cancer drug resistance. Seminars in cancer biology; 2018: Elsevier.

5. Lin WW, Ou GY, Lin JZ, Yi SJ, Yao WC, Pan HC, et al. Neuregulin 1 enhances cell adhesion molecule L1 like expression levels and promotes malignancy in human glioma. Oncol Lett. 2020;20(1):326-36.

6. Shirakawa Y, Hide T, Yamaoka M, Ito Y, Ito N, Ohta K, et al. Ribosomal protein S6 promotes stem‐like characters in glioma cells. Cancer science. 2020;111(6):2041-51.

7. Khabibov M, Garifullin A, Boumber Y, Khaddour K, Fernandez M, Khamitov F, et al. Signaling pathways and therapeutic approaches in glioblastoma multiforme. International journal of oncology. 2022;60(6):1-18.

8. Kanu OO, Hughes B, Di C, Lin N, Fu J, Bigner DD, et al. Glioblastoma multiforme oncogenomics and signaling pathways. Clinical medicine Oncology. 2009;3:CMO. S1008.

9. Macharia LW, Muriithi W, Heming CP, Nyaga DK, Aran V, Mureithi MW, et al. The genotypic and phenotypic impact of hypoxia microenvironment on glioblastoma cell lines. BMC cancer. 2021;21(1):1-20.

10. Chédeville AL, Lourdusamy A, Monteiro AR, Hill R, Madureira PA. Investigating glioblastoma response to hypoxia. Biomedicines. 2020;8(9):310.

11. Mikeladze MA, Dutysheva EA, Kartsev VG, Margulis BA, Guzhova IV, Lazarev VF. Disruption of the complex between GAPDH and Hsp70 sensitizes C6 glioblastoma cells to hypoxic stress. International journal of molecular sciences. 2021;22(4):1520.

12. Wang G, Wang J-J, Fu X-L, Guang R, To S-ST. Advances in the targeting of HIF-1α and future therapeutic strategies for glioblastoma multiforme. Oncology Reports. 2017;37(2):657-70.

13. Attwood KM, Robichaud A, Westhaver LP, Castle EL, Brandman DM, Balgi AD, et al. Raloxifene prevents stress granule dissolution, impairs translational control and promotes cell death during hypoxia in glioblastoma cells. Cell death & disease. 2020;11(11):1-18.

14. González-Mora AM, Garcia-Lopez P. Estrogen Receptors as Molecular Targets of Endocrine Therapy for Glioblastoma. International Journal of Molecular Sciences. 2021;22(22):12404.

15. Lee J, Troike K, Fodor Ra, Lathia JD. Unexplored functions of sex hormones in glioblastoma cancer stem cells. Endocrinology. 2022;163(3):bqac002.

16. Vander Heiden MG. Targeting cancer metabolism: a therapeutic window opens. Nature reviews Drug discovery. 2011;10(9):671-84.

17. Kathagen-Buhmann A, Schulte A, Weller J, Holz M, Herold-Mende C, Glass R, et al. Glycolysis and the pentose phosphate pathway are differentially associated with the dichotomous regulation of glioblastoma cell migration versus proliferation. Neuro-oncology. 2016;18(9):1219-29.

18. Shi Q, Hjelmeland AB, Keir ST, Song L, Wickman S, Jackson D, et al. A novel low‐molecular weight inhibitor of focal adhesion kinase, TAE226, inhibits glioma growth. Molecular Carcinogenesis: Published in cooperation with the University of Texas MD Anderson Cancer Center. 2007;46(6):488-96.

19. Park M-J, Kim M-S, Park I-C, Kang H-S, Yoo H, Park SH, et al. PTEN suppresses hyaluronic acid-induced matrix metalloproteinase-9 expression in U87MG glioblastoma cells through focal adhesion kinase dephosphorylation. Cancer research. 2002;62(21):6318-22.

20. Zheng Q, Han L, Dong Y, Tian J, Huang W, Liu Z, et al. JAK2/STAT3 targeted therapy suppresses tumor invasion via disruption of the EGFRvIII/JAK2/STAT3 axis and associated focal adhesion in EGFRvIII-expressing glioblastoma. Neuro-oncology. 2014;16(9):1229-43.

21. Wu YJ, Muldoon LL, Gahramanov S, Kraemer DF, Marshall DJ, Neuwelt EA. Targeting α V-integrins decreased metastasis and increased survival in a nude rat breast cancer brain metastasis model. Journal of neuro-oncology. 2012;110:27-36.

22. Iozzo RV, Sanderson RD. Proteoglycans in cancer biology, tumour microenvironment and angiogenesis. Journal of cellular and molecular medicine. 2011;15(5):1013-31.

23. Ahrens TD, Bang-Christensen SR, Jørgensen AM, Løppke C, Spliid CB, Sand NT, et al. The role of proteoglycans in cancer metastasis and circulating tumor cell analysis. Frontiers in cell and developmental biology. 2020;8:749.

24. Gheidari F, Arefian E, Adegani FJ, Kalhori MR, Seyedjafari E, Kabiri M, et al. miR-424 induces apoptosis in glioblastoma cells and targets AKT1 and RAF1 oncogenes from the ERBB signaling pathway. European journal of pharmacology. 2021;906:174273.

25. Clark PA, Iida M, Treisman DM, Kalluri H, Ezhilan S, Zorniak M, et al. Activation of multiple ERBB family receptors mediates glioblastoma cancer stem-like cell resistance to EGFR-targeted inhibition. Neoplasia. 2012;14(5):420-IN13.

26. Gheidari F, Arefian E, Saadatpour F, Kabiri M, Seyedjafari E, Teimoori-Toolabi L, et al. The miR-429 suppresses proliferation and migration in glioblastoma cells and induces cell-cycle arrest and apoptosis via modulating several target genes of ERBB signaling pathway. Molecular Biology Reports. 2022;49(12):11855-66.

27. Wee P, Wang Z. Epidermal growth factor receptor cell proliferation signaling pathways. Cancers. 2017;9(5):52.

28. Johnston ALM, Lun X, Rahn JJ, Liacini A, Wang L, Hamilton MG, et al. The p75 neurotrophin receptor is a central regulator of glioma invasion. PLoS biology. 2007;5(8):e212.

29. Liu H-J, Wang L, Kang L, Du J, Li S, Cui H-X. Sulforaphane-N-Acetyl-cysteine induces autophagy through activation of ERK1/2 in U87MG and U373MG cells. Cellular Physiology and Biochemistry. 2018;51(2):528-42.

30. Valdés-Rives SA, Casique-Aguirre D, Germán-Castelán L, Velasco-Velázquez MA, González-Arenas A. Apoptotic signaling pathways in glioblastoma and therapeutic implications. BioMed research international. 2017;2017.

31. Pawlowska E, Szczepanska J, Szatkowska M, Blasiak J. An interplay between senescence, apoptosis and autophagy in glioblastoma multiforme—role in pathogenesis and therapeutic perspective. International journal of molecular sciences. 2018;19(3):889.

32. Kälin RE, Glass R. APLN/APLNR signaling controls key pathological parameters of glioblastoma. Cancers. 2021;13(15):3899.

33. Harford-Wright E, Andre-Gregoire G, Jacobs KA, Treps L, Le Gonidec S, Leclair HM, et al. Pharmacological targeting of apelin impairs glioblastoma growth. Brain. 2017;140(11):2939-54.

34. Frisch A, Kälin S, Monk R, Radke J, Heppner FL, Kälin RE. Apelin controls angiogenesis-dependent glioblastoma growth. International journal of molecular sciences. 2020;21(11):4179.

35. Salam R, Saliou A, Bielle F, Bertrand M, Antoniewski C, Carpentier C, et al. Cellular senescence in malignant cells promotes tumor progression in mouse and patient Glioblastoma. Nature Communications. 2023;14(1):441.

36. Urbantat RM, Vajkoczy P, Brandenburg S. Advances in chemokine signaling pathways as therapeutic targets in glioblastoma. Cancers. 2021;13(12):2983.

37. Groblewska M, Litman-Zawadzka A, Mroczko B. The role of selected chemokines and their receptors in the development of gliomas. International journal of molecular sciences. 2020;21(10):3704.

38. Balmain A, Brown K. Oncogene activation in chemical carcinogenesis. Advances in cancer research. 1988;51:147-82.

39. Alibek K, Kakpenova A, Baiken Y. Role of infectious agents in the carcinogenesis of brain and head and neck cancers. Infectious agents and cancer. 2013;8(1):1-9.

40. Asad AS, Nicola Candia AJ, Gonzalez N, Zuccato CF, Seilicovich A, Candolfi M. The role of the prolactin receptor pathway in the pathogenesis of glioblastoma: what do we know so far? Expert Opinion on Therapeutic Targets. 2020;24(11):1121-33.

41. Asad AS, Nicola Candia AJ, Gonzalez N, Zuccato CF, Abt A, Orrillo SJ, et al. Prolactin and its receptor as therapeutic targets in glioblastoma multiforme. Scientific reports. 2019;9(1):1-16.

42. Turaga SM, Lathia JD. Adhering towards tumorigenicity: altered adhesion mechanisms in glioblastoma cancer stem cells. CNS oncology. 2016;5(4):251-9.

43. Yang H, Jin L, Sun X. A thirteen‑gene set efficiently predicts the prognosis of glioblastoma. Molecular Medicine Reports. 2019;19(3):1613-21.

44. Lu E, Gareev I, Yuan C, Liang Y, Sun J, Chen X, et al. The mechanisms of current platinum anticancer drug resistance in the glioma. Current Pharmaceutical Design. 2022;28(23):1863-9.

45. Brower JV, Clark PA, Lyon W, Kuo JS. MicroRNAs in cancer: glioblastoma and glioblastoma cancer stem cells. Neurochemistry international. 2014;77:68-77.

46. Vlahovic G, Fecci PE, Reardon D, Sampson JH. Programmed death ligand 1 (PD-L1) as an immunotherapy target in patients with glioblastoma. Oxford University Press; 2015. p. 1043-5.

47. Wang X, Guo G, Guan H, Yu Y, Lu J, Yu J. Challenges and potential of PD-1/PD-L1 checkpoint blockade immunotherapy for glioblastoma. Journal of Experimental & Clinical Cancer Research. 2019;38:1-13.

48. Maghrouni A, Givari M, Jalili-Nik M, Mollazadeh H, Bibak B, Sadeghi MM, et al. Targeting the PD-1/PD-L1 pathway in glioblastoma multiforme: Preclinical evidence and clinical interventions. International immunopharmacology. 2021;93:107403.

49. Litak J, Mazurek M, Grochowski C, Kamieniak P, Roliński J. PD-L1/PD-1 axis in glioblastoma multiforme. International journal of molecular sciences. 2019;20(21):5347.

50. Morford LA, Elliott LH, Carlson SL, Brooks WH, Roszman TL. T cell receptor-mediated signaling is defective in T cells obtained from patients with primary intracranial tumors. Journal of immunology (Baltimore, Md: 1950). 1997;159(9):4415-25.

51. Land CA, Musich PR, Haydar D, Krenciute G, Xie Q. Chimeric antigen receptor T-cell therapy in glioblastoma: charging the T cells to fight. Journal of translational medicine. 2020;18:1-13.

52. Woroniecka KI, Rhodin KE, Chongsathidkiet P, Keith KA, Fecci PE. T-cell Dysfunction in Glioblastoma: Applying a New FrameworkT-cell Dysfunction in Glioblastoma. Clinical Cancer Research. 2018;24(16):3792-802.

53. Soukhtanloo M, Mohtashami E, Maghrouni A, Mollazadeh H, Mousavi SH, Roshan MK, et al. Natural products as promising targets in glioblastoma multiforme: a focus on NF-κB signaling pathway. Pharmacological reports. 2020;72:285-95.

54. Chattopadhyay N, Evliyaoglu C, Heese O, Carroll R, Sanders J, Black P, et al. Regulation of secretion of PTHrP by Ca2+-sensing receptor in human astrocytes, astrocytomas, and meningiomas. American Journal of Physiology-Cell Physiology. 2000;279(3):C691-C9.

55. Chakravarti B, Dwivedi SKD, Mithal A, Chattopadhyay N. Calcium-sensing receptor in cancer: good cop or bad cop? Endocrine. 2009;35:271-84.

56. Ferri A, Nencini M, Casciati A, Cozzolino M, Angelini DF, Longone P, et al. Cell death in amyotrophic lateral sclerosis: interplay between neuronal and glial cells. The FASEB journal. 2004;18(11):1261-3.

57. Benigni M, Ricci C, Jones AR, Giannini F, Al-Chalabi A, Battistini S. Identification of miRNAs as potential biomarkers in cerebrospinal fluid from amyotrophic lateral sclerosis patients. Neuromolecular medicine. 2016;18:551-60.

58. Qin G, Hu B, Li X, Li R, Meng Y, Wang Y, et al. Identification of key differentially expressed transcription factors in glioblastoma. Journal of Oncology. 2020;2020.

59. Cao J-Y, Guo Q, Guan G-F, Zhu C, Zou C-Y, Zhang L-Y, et al. Elevated lymphocyte specific protein 1 expression is involved in the regulation of leukocyte migration and immunosuppressive microenvironment in glioblastoma. Aging (Albany NY). 2020;12(2):1656.

60. Lazarev VF, Nikotina AD, Mikhaylova ER, Nudler E, Polonik SG, Guzhova IV, et al. Hsp70 chaperone rescues C6 rat glioblastoma cells from oxidative stress by sequestration of aggregating GAPDH. Biochemical and biophysical research communications. 2016;470(3):766-71.

61. Van Brocklyn JR. Sphingolipid signaling pathways as potential therapeutic targets in gliomas. Mini reviews in medicinal chemistry. 2007;7(10):984-90.

62. Tea MN, Poonnoose SI, Pitson SM. Targeting the sphingolipid system as a therapeutic direction for glioblastoma. Cancers. 2020;12(1):111.

63. Akhavan D, Cloughesy TF, Mischel PS. mTOR signaling in glioblastoma: lessons learned from bench to bedside. Neuro-oncology. 2010;12(8):882-9.

64. Jung E, de los Reyes V AA, Pumares KJA, Kim Y. Strategies in regulating glioblastoma signaling pathways and anti-invasion therapy. PLoS One. 2019;14(4):e0215547.

65. Zheng X, Li W, Xu H, Liu J, Ren L, Yang Y, et al. Sinomenine ester derivative inhibits glioblastoma by inducing mitochondria-dependent apoptosis and autophagy by PI3K/AKT/mTOR and AMPK/mTOR pathway. Acta Pharmaceutica Sinica B. 2021;11(11):3465-80.

66. Thomas L, Florio T, Perez-Castro C. Extracellular vesicles loaded miRNAs as potential modulators shared between glioblastoma, and Parkinson’s and Alzheimer’s diseases. Frontiers in Cellular Neuroscience. 2020;14:590034.

67. Liu T, Ren D, Zhu X, Yin Z, Jin G, Zhao Z, et al. Transcriptional signaling pathways inversely regulated in Alzheimer's disease and glioblastoma multiform. Scientific reports. 2013;3(1):3467.

68. Candido S, Lupo G, Pennisi M, Basile MS, Anfuso CD, Petralia MC, et al. The analysis of miRNA expression profiling datasets reveals inverse microRNA patterns in glioblastoma and Alzheimer's disease. Oncology Reports. 2019;42(3):911-22.

69. Sánchez-Valle J, Tejero H, Ibáñez K, Portero JL, Krallinger M, Al-Shahrour F, et al. A molecular hypothesis to explain direct and inverse co-morbidities between Alzheimer’s Disease, Glioblastoma and Lung cancer. Scientific reports. 2017;7(1):1-12.

70. Sivori S, Parolini S, Marcenaro E, Castriconi R, Pende D, Millo R, et al. Involvement of natural cytotoxicity receptors in human natural killer cell-mediated lysis of neuroblastoma and glioblastoma cell lines. Journal of neuroimmunology. 2000;107(2):220-5.

71. Kondo S, Yin D, Takeuchi J, Morimura T, Miyatake S, Nakatsu S, et al. Tumour necrosis factor-α induces an increase in susceptibility of human glioblastoma U87-MG cells to natural killer cell-mediated lysis. British journal of cancer. 1994;69(4):627-32.

72. Ge Y-F, Sun J, Jin C-J, Cao B-Q, Jiang Z-F, Shao J-F. AntagomiR-27a targets FOXO3a in glioblastoma and suppresses U87 cell growth in vitro and in vivo. Asian pacific journal of cancer prevention. 2013;14(2):963-8.

73. Sunayama J, Sato A, Matsuda K-I, Tachibana K, Watanabe E, Seino S, et al. FoxO3a functions as a key integrator of cellular signals that control glioblastoma stem-like cell differentiation and tumorigenicity. Stem cells. 2011;29(9):1327-37.

74. Aroui S, Dardevet L, Najlaoui F, Kammoun M, Laajimi A, Fetoui H, et al. PTEN-regulated AKT/FoxO3a/Bim signaling contributes to Human cell glioblastoma apoptosis by platinum-maurocalcin conjugate. The international journal of biochemistry & cell biology. 2016;77:15-22.

75. Ciechomska IA, Gielniewski B, Wojtas B, Kaminska B, Mieczkowski J. EGFR/FOXO3a/BIM signaling pathway determines chemosensitivity of BMP4-differentiated glioma stem cells to temozolomide. Experimental & molecular medicine. 2020;52(8):1326-40.

76. Jarabo P, de Pablo C, Herranz H, Martín FA, Casas-Tintó S. Insulin signaling mediates neurodegeneration in glioma. Life Science Alliance. 2021;4(3).

77. Tirrò E, Massimino M, Romano C, Martorana F, Pennisi MS, Stella S, et al. Prognostic and therapeutic roles of the insulin growth factor system in glioblastoma. Frontiers in Oncology. 2021;10:612385.

78. Gong Y, Ma Y, Sinyuk M, Loganathan S, Thompson RC, Sarkaria JN, et al. Insulin-mediated signaling promotes proliferation and survival of glioblastoma through Akt activation. Neuro-oncology. 2015;18(1):48-57.

79. Zhou M, Sareddy GR, Li M, Liu J, Luo Y, Venkata PP, et al. Estrogen receptor beta enhances chemotherapy response of GBM cells by down regulating DNA damage response pathways. Scientific reports. 2019;9(1):6124.

80. Sareddy GR, Pratap UP, Venkata PP, Zhou M, Alejo S, Viswanadhapalli S, et al. Activation of estrogen receptor beta signaling reduces stemness of glioma stem cells. Stem Cells. 2021;39(5):536-50.

81. Qazi H, Shi Z-D, Tarbell JM. Fluid shear stress regulates the invasive potential of glioma cells via modulation of migratory activity and matrix metalloproteinase expression. PloS one. 2011;6(5):e20348.

82. Krizanac-Bengez L, Mayberg MR, Janigro D. The cerebral vasculature as a therapeutic target for neurological disorders and the role of shear stress in vascular homeostatis and pathophysiology. Neurological research. 2004;26(8):846-53.

83. Chung J, Kim KH, An SH, Lee S, Lim B-K, Kang SW, et al. Coxsackievirus and adenovirus receptor mediates the responses of endothelial cells to fluid shear stress. Experimental & Molecular Medicine. 2019;51(11):1-15.

84. Zanotto-Filho A, Braganhol E, Klafke K, Figueiró F, Terra SR, Paludo FJ, et al. Autophagy inhibition improves the efficacy of curcumin/temozolomide combination therapy in glioblastomas. Cancer letters. 2015;358(2):220-31.

85. Simpson JE, Gammoh N. The impact of autophagy during the development and survival of glioblastoma. Open Biology. 2020;10(9):200184.

86. Li Z, Wang H, Eyler CE, Hjelmeland AB, Rich JN. Turning cancer stem cells inside out: an exploration of glioma stem cell signaling pathways. Journal of Biological Chemistry. 2009;284(25):16705-9.

87. Changbin Zhu M, Mustafa D, Dekker L, Kros JM, Cheng C. Proteome analysis of the CECR1 mediated response in tumor associated macrophages identifies key pathways and molecules in the immune response regulation of glioma. On the immune regulation of glioma angiogenesis. 2017:121.

88. Liu M, Xu Z, Du Z, Wu B, Jin T, Xu K, et al. The identification of key genes and pathways in glioma by bioinformatics analysis. Journal of Immunology Research. 2017;2017.

89. Yang Ja, Yang Q. Identification of core genes and screening of potential targets in glioblastoma multiforme by integrated bioinformatic analysis. Frontiers in Oncology. 2021;10:615976.

90. Ou A, Ott M, Fang D, Heimberger AB. The role and therapeutic targeting of JAK/STAT signaling in glioblastoma. Cancers. 2021;13(3):437.

91. Tu Y, Zhong Y, Fu J, Cao Y, Fu G, Tian X, et al. Activation of JAK/STAT signal pathway predicts poor prognosis of patients with gliomas. Medical Oncology. 2011;28:15-23.

92. Ryskalin L, Biagioni F, Busceti CL, Lazzeri G, Frati A, Fornai F. The multi-faceted effect of curcumin in glioblastoma from rescuing cell clearance to autophagy-independent effects. Molecules. 2020;25(20):4839.

93. Tang Q, Len Q, Liu Z, Wang W. Overexpression of miR‐22 attenuates oxidative stress injury in diabetic cardiomyopathy via Sirt 1. Cardiovascular therapeutics. 2018;36(2):e12318.

94. Bao Z, Chen K, Krepel S, Tang P, Gong W, Zhang M, et al. High glucose promotes human glioblastoma cell growth by increasing the expression and function of chemoattractant and growth factor receptors. Translational Oncology. 2019;12(9):1155-63.

95. Shao Y, Li M, Yu Q, Gong M, Wang Y, Yang X, et al. CircRNA CDR1as promotes cardiomyocyte apoptosis through activating hippo signaling pathway in diabetic cardiomyopathy. European Journal of Pharmacology. 2022;922:174915.

96. Kim SJ, Park SJ, Park J, Cho HJ, Shim J-K, Seon J, et al. Dual inhibition of CPT1A and G6PD suppresses glioblastoma tumorspheres. Journal of Neuro-Oncology. 2022:1-13.

97. Gutmann DH, Saporito-Irwin S, DeClue JE, Wienecke R, Guha A. Alterations in the rap1 signaling pathway are common in human gliomas. Oncogene. 1997;15(13):1611-6.

98. Guo D, Bell EH, Chakravarti A. Lipid metabolism emerges as a promising target for malignant glioma therapy. CNS oncology. 2013;2(3):289-99.

99. Kou Y, Geng F, Guo D. Lipid metabolism in glioblastoma: from de novo synthesis to storage. Biomedicines. 2022;10(8):1943.

100. Sarmiento-Salinas FL, Perez-Gonzalez A, Acosta-Casique A, Ix-Ballote A, Diaz A, Treviño S, et al. Reactive oxygen species: Role in carcinogenesis, cancer cell signaling and tumor progression. Life Sciences. 2021;284:119942.

101. Afshari AR, Jalili-Nik M, Soukhtanloo M, Ghorbani A, Sadeghnia HR, Mollazadeh H, et al. Auraptene-induced cytotoxicity mechanisms in human malignant glioblastoma (U87) cells: role of reactive oxygen species (ROS). EXCLI journal. 2019;18:576.

102. Chiu W-T, Shen S-C, Chow J-M, Lin C-W, Shia L-T, Chen Y-C. Contribution of reactive oxygen species to migration/invasion of human glioblastoma cells U87 via ERK-dependent COX-2/PGE2 activation. Neurobiology of disease. 2010;37(1):118-29.

103. Memmel S, Sisario D, Zöller C, Fiedler V, Katzer A, Heiden R, et al. Migration pattern, actin cytoskeleton organization and response to PI3K-, mTOR-, and Hsp90-inhibition of glioblastoma cells with different invasive capacities. Oncotarget. 2017;8(28):45298.

104. Avci NG, Ebrahimzadeh-Pustchi S, Akay YM, Esquenazi Y, Tandon N, Zhu J-J, et al. NF-κB inhibitor with Temozolomide results in significant apoptosis in glioblastoma via the NF-κB (p65) and actin cytoskeleton regulatory pathways. Scientific Reports. 2020;10(1):13352.

105. Tsvetkov AS, Miller J, Arrasate M, Wong JS, Pleiss MA, Finkbeiner S. A small-molecule scaffold induces autophagy in primary neurons and protects against toxicity in a Huntington disease model. Proceedings of the National Academy of Sciences. 2010;107(39):16982-7.

106. Houghton P, Thimmaiah K, Easton J. Substituted phenoxazines and acridones as inhibitors of AKT. Google Patents; 2006.

107. Bingxiang X, Panxing W, Lu F, Xiuyou Y, Chao D. A prognostic model for brain glioma patients based on 9 signature glycolytic genes. BioMed research international. 2021;2021.

108. Sharpe MA, Ijare OB, Baskin DS, Baskin AM, Baskin BN, Pichumani K. The leloir cycle in glioblastoma: Galactose scavenging and metabolic remodeling. Cancers. 2021;13(8):1815.
