# Supplementary material for: Molecular signature to predict quality of life and survival with glioblastoma using Multiview omics model
Source: PLoS One. 2023 Nov 16;18(11):e0287448. doi: 10.1371/journal.pone.0287448 (PMC10653472; doi:10.1371/journal.pone.0287448)
Supplement: Attachment — Submitted filename: Response to Reviewers.docx [file pone.0287448.s005.docx]

**Respond to comments:**

3. Thank you for stating the following in the Acknowledgments Section of your manuscript: 
"We thank our colleagues at Birmingham University and KAIMRC for support, comments, and critique to the project: Danesh Moradigaravand, Justina Žurauskienė, Mamoon Rashid, and Jean-Baptiste Cazier. We thank the King Abdullah International Medical Research Center (KAMRC) for sponsoring this work under protocol No. RC13/258/R and NRC.21R.093.03."

We note that you have provided funding information that is not currently declared in your Funding Statement. However, funding information should not appear in the Acknowledgments section or other areas of your manuscript. We will only publish funding information present in the Funding Statement section of the online submission form. 
Please remove any funding-related text from the manuscript and let us know how you would like to update your Funding Statement.

As requested, funding information has been removed.

The funding statement as follow: "King Abdullah International Medical Research Center (KAMRC) sponsored this work under protocol No. RC13/258/R and and NRC.21R.093.03.
The funders had no role in study design, data collection and analysis, decision to publish, or preparation of the manuscript. "

4. Thank you for stating the following in your Competing Interests section:  
"All authors report no competing interests."

Please complete your Competing Interests on the online submission form to state any Competing Interests. If you have no competing interests, please state "The authors have declared that no competing interests exist.", as detailed online in our guide for authors at <http://journals.plos.org/plosone/s/submit-now>

The manuscript has been updated as requested

5.  In your Data Availability statement, you have not specified where the minimal data set underlying the results described in your manuscript can be found. PLOS defines a study's minimal data set as the underlying data used to reach the conclusions drawn in the manuscript and any additional data required to replicate the reported study findings in their entirety. All PLOS journals require that the minimal data set be made fully available. For more information about our data policy, please see <http://journals.plos.org/plosone/s/data-availability>.

It has been stated in the manuscript under “Data collection and pre-processing” the original source TCGA website and the links for all the data extracted. Any bioinformatician should be able to extract the data using RStudio software.

6. We note that Figure 3b and 3c in your submission contain copyrighted images. All PLOS content is published under the Creative Commons Attribution License (CC BY 4.0), which means that the manuscript, images, and Supporting Information files will be freely available online, and any third party is permitted to access, download, copy, distribute, and use these materials in any way, even commercially, with proper attribution. For more information, see our copyright guidelines: <http://journals.plos.org/plosone/s/licenses-and-copyright>.
 
We require you to either (1) present written permission from the copyright holder to publish these figures specifically under the CC BY 4.0 license, or (2) remove the figures from your submission:

Those figures have been moved to Fig 4b and 4c

For Fig.4b we have remade the figure in power point and since the data is our data no need permission at this point.

For Fig. 4c the permission states, “All data and download files in STRING are freely available under a 'Creative Commons BY 4.0' license. When using the data, please provide appropriate credit — and inform users of any changes or additions that you might have made to the data.” Which was already granted at the following link.

<https://string-db.org/cgi/access?sessionId=bR32tJ1PGdkQ&footer_active_subpage=licensing>

7. Please include a copy of Table 2 which you refer to in your text on page 14.

(DONE) Table 2 has been added to page 18

8. Please include captions for your Supporting Information files at the end of your manuscript, and update any in-text citations to match accordingly. Please see our Supporting Information guidelines for more information: http://journals.plos.org/plosone/s/supporting-information.

The manuscript has been updated

**Comments to the Author**

1. Is the manuscript technically sound, and do the data support the conclusions?

The manuscript must describe a technically sound piece of scientific research with data that supports the conclusions. Experiments must have been conducted rigorously, with appropriate controls, replication, and sample sizes. The conclusions must be drawn appropriately based on the data presented.

Reviewer #1: Partly

Reviewer #2: Partly

2. Has the statistical analysis been performed appropriately and rigorously?

Reviewer #1: I Don't Know

Reviewer #2: I Don't Know

3. Have the authors made all data underlying the findings in their manuscript fully available?

The [PLOS Data policy](https://protect2.fireeye.com/v1/url?k=f83aa4b5-9abf5b48-f839b3f2-c400ad4e22b2-083da358b1ec7f5f&q=1&e=f02c8d9a-055d-4322-9528-d96f3f00f2c7&u=http%3A%2F%2Fwww.plosone.org%2Fstatic%2Fpolicies.action%23sharing) requires authors to make all data underlying the findings described in their manuscript fully available without restriction, with rare exception (please refer to the Data Availability Statement in the manuscript PDF file). The data should be provided as part of the manuscript or its supporting information, or deposited to a public repository. For example, in addition to summary statistics, the data points behind means, medians and variance measures should be available. If there are restrictions on publicly sharing data—e.g. participant privacy or use of data from a third party—those must be specified.

Reviewer #1: No

Reviewer #2: Yes

As mentioned in the manuscript, all data used in this project are available as tar.gz file. See methods section page 5 line 75-76. “ To download all data as a tar.gz file use the link (<https://cbioportal-datahub.s3.amazonaws.com/gbm_tcga.tar.gz>/ accessed on 1 June 2022).”

4. Is the manuscript presented in an intelligible fashion and written in standard English?

PLOS ONE does not copyedit accepted manuscripts, so the language in submitted articles must be clear, correct, and unambiguous. Any typographical or grammatical errors should be corrected at revision, so please note any specific errors here.

Reviewer #1: Yes

Reviewer #2: Yes

5. Review Comments to the Author

Please use the space provided to explain your answers to the questions above. You may also include additional comments for the author, including concerns about dual publication, research ethics, or publication ethics. (Please upload your review as an attachment if it exceeds 20,000 characters)

Reviewer #1: In this study, the authors proposed a bioinformatics analysis to predict quality of life and survival with glioblastoma using multi-omics model. Although the performance seems promising, some major points should be addressed as follows:

Thank you very much

**Reviewer #1:**

1. All analyses were conducted on TCGA public data without any validation.

Actually, we conducted two steps of validations: Internal validation on 20% of TCGA data (test dataset) which was not analyzed in the original analysis, (see the model performance in supplementary figure 1, figure 2, and figure 6d). External validation where we applied the predicted molecular signatures on CPTAC data, (see table 4). Those were 2 different validation approaches.

2. At least mutations in the driver genes should be investigated.

(We added a part for mutations in driver genes. See supplementary figure 3, See discussion page 29 line 533-537)

3. It is unclear why iRF has been used as a feature selection method rather than the others. This step even can be done using traditional approaches i.e., univariate, statistical tests, etc. Thus, the authors should describe this part clearly or have a baseline comparison on it.

Manuscript updated and quoted below, See discussion page 24-25 line 424-434

“ Compared to other machine learning methods for analyzing integrative omics data, iRF has several advantages. Firstly, it uses a recursive feature elimination approach to iteratively eliminate the least informative features to enhance model accuracy and stability in high-dimensional datasets (18). Secondly, iRF is designed to identify high-order interactions between features which is essential for integrative omics data where many biological processes involve interactions among multiple molecular features from multiple sources. This makes iRF an excellent method for biological and biomedical applications. Thirdly, iRF has been shown to handle noisy data. Building a new random forest at each iteration eliminates the effect of correlated variables and noisy data on feature importance. The invariance of decision trees also ensures less bias due to the signal-to-noise ratio among biological replicates, a significant concern in omics data analysis. ”

4. The authors reported ROC AUC and PRC but they did not show ROC curves, PR curves.

The ROC and PR curves have been added (See supplementary figure 1)

5. More references on bioinformatics workflow i.e., PMID: 35851932, PMID: 34572330 should be added to attract a broader readership.

The references have been added, and relevant comments added, See introduction page 4 line 54-55

6. Literature review is insufficient; the authors may improve it using some related works on bioinformatics-based glioblastoma.

Literature review has been updated, See introduction page 3-4 line 38-47)

7. Quality of figures should be improved.

Higher quality figures have been added

**Reviewer #2:**

1. The materials and methods section of this paper needs to be presented much before the results sections of the paper as it makes it difficult to interpret the results without understanding the mechanics of all the algorithms involved. The description of methods in the results section is breif and vague and leaves the readers with lots of questions regarding the methods. I would suggest the authors to explain their analysis pipeline well in the introduction section, if they are unable to move the methods section prior to the results. Keeping the diverse background of the PLOS ONE readership in mind, it is important to note that not all readers will be aware of methods used in this paper, prior to reading the methods section.

The methods section has been moved to before the results and after the introduction. More explanation was added.

2. The abstract and introduction introduces the topic of integrative omics, the authors need to clarify the meaning of this term very lucidly in these early sections of the paper. For example, they can state that it integrates omics data with patient-level clinical data to identify biomarkers or molecular signatures.

Abstract updated line 4-6

Manuscript updated and quoted below, See introduction page 3-4 line 29-37 and 47-49)

“ Integrative omics is a promising approach to unlocking new insights into the mechanisms underlying GBM prognosis and identifying new biomarkers and therapeutic targets(6). Integrative omics approaches combine different types of omics data, such as genomics, transcriptomics, proteomics, and metabolomics, to obtain a more comprehensive understanding of biological processes and pathways in complex biological systems including cancers(6). Using the integrative omics approach could reveal previously unknown connections and relationships between molecules, pathways, and biological processes in GBM prognosis. In addition, integrating omics data with patient-level clinical data to identify molecular signatures or biomarkers can help predict disease risk, prognosis, and treatment response, which can be particularly valuable in personalized medicine and individualized treatment plans. Several studies have demonstrated the effectiveness of integrative omics and machine learning approaches in improving our understanding of different cancers including GBM(7-9). One study showed that integrating gene expression and copy number data improved the accuracy of GBM subtype classification(10). Another study showed that integrating single nucleotide polymorphism (SNP), DNA copy number, DNA methylation, mRNA expression, and clinical data identified new GBM subtypes with distinct clinical outcomes(11). Furthermore, integrative omics approaches have also been used to identify potential therapeutic targets for GBM(12, 13). Despite the growing interest in this approach, integrative omics is still missing in QOL studies.”

“ In this study, we integrated gene expression and proteomics data with GBM patients’ clinical data including KPS and overall survival (OS) to predict the molecular signatures associated with enhanced GBM patients’ survival and QOL. ”

3.The authors to explain the KPS scale in detail in the introduction section. For example what does it mean to say QOL >= 80 KPS.

Manuscript updated and quoted below, See introduction page 4 line 56-63

“ Our goal was to identify molecular signatures for GBM patients who survived ≥ 2 years with a QOL ≥ 80 KPS. We intended to differentiate between short-term (≤ 6 months) and long-term (≥ 2 years) GBM survival, as well as between high KPS ≥ 80, and low KPS ≤ 60. KPS system represents three main categories: KPS ≤ 80 which represents the ability to work and carry on normally with no special care needed; KPS ≤ 60, which requires occasional assistance and impairs work but allows self-care, and KPS ≤ 40, which renders patients unable to look after themselves, necessitates hospital care, and the disease progresses rapidly. This means QOL ≥ 80 KPS represents a quality of life equal or better than KPS group 80 which represents the ability to work and carry on normal tasks with no special care needed. ”

4. Please explain why iRF method was used, how it is relevant to your dataset, and what other methods did you consider.

Manuscript updated and quoted below, See discussion page 24-25 lines 420-422 and 426-438

“ We used the iRF algorithm(18) for feature selection in integrative omics data. This method is preferred because it identifies stable and robust high-order interactions between features, maintains high accuracy, and is interpretable. ”

“ iRF has several advantages. Firstly, it uses a recursive feature elimination approach to iteratively eliminate the least informative features to enhance model accuracy and stability in high-dimensional datasets (18). Secondly, iRF is designed to identify high-order interactions between features which is essential for integrative omics data where many biological processes involve interactions among multiple molecular features from multiple sources. This makes iRF an excellent method for biological and biomedical applications. Thirdly, iRF has been shown to handle noisy data. Building a new random forest at each iteration eliminates the effect of correlated variables and noisy data on feature importance. The invariance of decision trees also ensures less bias due to the signal-to-noise ratio among biological replicates, a significant concern in omics data analysis. It also deals with imbalanced data without affecting model performance. The iRF algorithm is less prone to overfitting than other machine learning approaches because it utilizes much smaller decision trees, mitigating the problem of overfitting in high-dimensional integrative omics data. ”

5.In the result sections , page 10, paragraph 1: The authors should explicitly mention that they are using iRF to classify between long and short term GBM survival, if not please provide more clarification of the objective here.

Manuscript updated and quoted below, See results section page 10-11 line 192, and 212

“ We used the iRF approach to predict important features that classify patients who lived ≥ 2 years”

“ The significant features we obtained from the MiRF model are presented in Table 1.”

6.In the results sections the authors have presented AUPR scores with confidence intervals multiple times, they need to provide some qualitative explanation or significance of the results to the readers here. Also the authors need to clarify the parameters K=5 and Ba =10, and how these values were determined.

Manuscript updated for qualitative explanation, example quoted below, (See results section page 11 lines (197-199), (205-208), (211-212)

“ respectively, for K = 5 and R = 100 (Fig 2a). These results show that our models have relatively acceptable performance, as an AUC value closer to 1 indicates nonrandomized prediction.”

For K and R, Figure 1 explain the usage of K and Ba ( Ba replaced by R). K is the number of iterations. The R parameter represents the number of runs we have done for each model using bootstrapped samples. Both are explained in the method and since the methods have been moved to the top after introduction section, we assumed no further explanation is needed. The numbers can be increased to increase significance but practically increasing the numbers will increase the computing time from hours to days per cycle. Any researcher repeating the model would face this issue unless he own a super-computer. Statistically, the numbering is also subjective somehow but this equation would help K= (aggregated interactions“50” divided by 10-fold cross-validation)=5 See methods page 7 line is 118-123.

7. The authors state that "Additionally, we used iRF to recover the important interactions between these features." Can they explain what is the meaning of the term "important interactions", what is the process involved here in determining these interactions ? Is this a pairwise score between features or is a feature importance metric ?

Manuscript updated and quoted below, See discussion (page 24, line 428-431, and page 26 lines 471-479)

“Secondly, iRF is designed to identify high-order interactions between features which is essential for integrative omics data where many biological processes involve interactions among multiple molecular features from multiple sources.”

“ It employs a feature importance metric to identify important and significant interactions between features. The feature importance metric assigns a score (mean decrease in Gini impurity) to each feature that measures its contribution to prediction as part of an interaction. The feature importance score is determined by two factors: feature impact which reflects a feature's individual effect on the outcome, and feature importance with respect to interaction which measures the contribution of a feature to complex interactions. This feature importance metric enables iRF to provide more accurate and interpretable predictions while accounting for the complexity of the underlying interactions between features.”

8.Please explain the meaning of stability scores for example in the line "Recovered interactions are shown in Figure 2, which include stability scores for both OS ≥ 2years (Fig. 2a) and QOL ≥ 80 KPS (Fig. 2b)."

Manuscript updated and quoted below, (See discussion page 26 line 479-482)

“ We also calculated a stability score which is a measure of the robustness of the discovered interactions. The stability score provides an estimate of the probability that a particular interaction is consistently recovered across different bootstrapped samples of the training dataset.”

9.In the functional annotation and interaction analysis section, are any of the pathways the author found previously implicated in GBM or other cancers increased survival and positive clinical outcomes , i.e. OS>=2 and QOL >= 80 KPS ? Or are they new to the GBM literature ?

Yes they are, see supplementary table 2 a and b, We introduced all biological functions and pathways resulted from our functional analysis. In term of pathways, supplementary table 2b has all references for the already known pathways, the new pathways have no references. Also see discussion page 27-29 lines 492-539)

“ In addition to the well-known biological functions and pathways, we identified a novel association of neurodegenerative, cardiac, vascular, viral, bacterial, and other cancers pathways with GBM (S2a and S2b Tables).”

10. The authors need to clarify how the elastic net penalties were calculated or assigned for CPH regression model. (methods)

This function automated through the `glmnet` package in R software which provides a function `glmnet` for fitting a Cox proportional hazards model with elastic net regularization. The elastic net penalty parameter is determined by cross-validation on the training data.

Here's how the elastic net penalty is determined:

1. The input data is split into training and validation sets.

2. The `glmnet` function fits a Cox proportional hazards model with elastic net regularization over a range of possible penalty parameters `lambda`.

3. For each value of `lambda`, a 10-fold cross-validation is performed on the training data to estimate the Harrell C-index associated with that value of `lambda` using `cv.glmnet` function.

4. The value of `lambda` that maximizes the Harrell C-index value is selected as the optimal value for the elastic net penalty.

5. A final model is fit using the optimal value of `lambda` on the entire training dataset. The model's performance is then evaluated on the validation dataset.

In summary, the `glmnet` function in `glmnet` R package determines the elastic net penalty for the Cox proportional hazards model by cross validating the performance of the model over a range of penalty parameters, and selecting the value of the penalty parameter that results in the best performance on the validation dataset.)

In the manuscript: “ “glmnet” R package (Jerome Friedman et al, 2010) (24) was used to fit the regularized CPH regression model.” (See methods section page 9 line 158-164.)

11. Please explain if the PCA validation was done on the entire dataset or on a specific hold out or test data set ? This needs to be clearly explained as validation must be carried out on a dataset on which the models were not trained. (methods)

Actually, the PCA was done on the entire dataset including trained (represent 80%) and untrained (represent 20%) of dataset, our intention was to visualize the effect of those MiRF predicted molecular features to distinguish between different groups.

Manuscript updated and quoted below, See methods section page 10 line 173-175

“To visualize the effect of the MiRF-predicted molecular signatures on classification between OS ≤ 6 months and OS ≥ 2 years and KPS ≥ 80 and KPS ≤ 60 groups, we calculated PCA components of the predicted signatures using the entire specific data we extracted from TCGA.”

12.Please cite the R Studio, here is a link on how to do it: https://psyteachr.github.io/quant-fun-v2/citing-r-rstudio.html

Manuscript updated, (See methods page 5 line 76-78)

“ Data processing was performed using R Statistical Software (R Core Team, 2021), and RStudio (Rstudio Team, 2022.02.3).”

13.Figure 1 has only labels 'a' and 'b', the other labels mentioned in the figure caption are missing.

Sorry about that, Manuscript updated

14. The overall English language should be reviewed thoroughly, there are typos in the manuscript, the gene names should be italicized, and figure resolutions need improvement.

Manuscript updated
